# Genetic regulation of volatile production in two melon introgression line collections with contrasting ripening behavior

**DOI:** 10.1093/hr/uhae020

**Published:** 2024-01-16

**Authors:** Carlos Mayobre, Miguel Santo Domingo, Elif Nur Özkan, Andrés Fernández-Borbolla, Javier Ruiz-Lasierra, Jordi Garcia-Mas, Marta Pujol

**Affiliations:** Centre for Research in Agricultural Genomics (CRAG) CSIC-IRTA-UAB-UB, Edifici CRAG, Campus UAB, 08193 Bellaterra, Barcelona, Spain; Centre for Research in Agricultural Genomics (CRAG) CSIC-IRTA-UAB-UB, Edifici CRAG, Campus UAB, 08193 Bellaterra, Barcelona, Spain; Centre for Research in Agricultural Genomics (CRAG) CSIC-IRTA-UAB-UB, Edifici CRAG, Campus UAB, 08193 Bellaterra, Barcelona, Spain; Centre for Research in Agricultural Genomics (CRAG) CSIC-IRTA-UAB-UB, Edifici CRAG, Campus UAB, 08193 Bellaterra, Barcelona, Spain; Centre for Research in Agricultural Genomics (CRAG) CSIC-IRTA-UAB-UB, Edifici CRAG, Campus UAB, 08193 Bellaterra, Barcelona, Spain; Centre for Research in Agricultural Genomics (CRAG) CSIC-IRTA-UAB-UB, Edifici CRAG, Campus UAB, 08193 Bellaterra, Barcelona, Spain; Institut de Recerca i Tecnologia Agroalimentàries (IRTA), Edifici CRAG, Campus UAB, 08193 Bellaterra, Barcelona, Spain; Centre for Research in Agricultural Genomics (CRAG) CSIC-IRTA-UAB-UB, Edifici CRAG, Campus UAB, 08193 Bellaterra, Barcelona, Spain; Institut de Recerca i Tecnologia Agroalimentàries (IRTA), Edifici CRAG, Campus UAB, 08193 Bellaterra, Barcelona, Spain

## Abstract

The importance of melon aroma in determining fruit quality has been highlighted in recent years. The fruit volatile profile is influenced by the type of fruit ripening. Non-climacteric fruits contain predominantly aldehydes, while climacteric fruits mainly produce esters. Several genes have been described to participate in volatile organic compounds (VOCs) biosynthesis pathways, but knowledge in this area is still incomplete. In this work we analysed the volatile profile of two reciprocal Introgression Line (IL) collections generated from a cross between ‘Piel de Sapo' (PS) and ‘Védrantais’ (VED) melons, differing in their aroma profile and ripening behaviour. SPME GC–MS was performed to identify genes responsible for VOCs formation. More than 1000 QTLs for many volatiles were detected taken together both populations. Introgressions on chromosomes 3, 5, 6, 7 and 8 modified ester-aldehyde balance and were correlated to ripening changes in both genetic backgrounds. Some previously identified QTLs for fruit ripening might be involved in these phenotypes, such as *ETHQV8.1* on chromosome 8 and *ETHQV6.3* on chromosome 6. PS alleles on chromosomes 2, 6, 10 and 11 were found to increase ester content when introgressed in VED melons. Terpenes showed to be affected by several genomic regions not related to ripening. In addition, several candidate genes have been hypothesized to be responsible for some of the QTLs detected. The analysis of volatile compounds in two reciprocal IL collections has increased our understanding of the relationship between ripening and aroma and offers valuable plant material to improve food quality in melon breeding programs.

## Introduction

Melon flavor is one of the most important fruit quality traits for consumers. Fruit taste depends on the volatile organic compounds (VOCs) composition, texture, sugars, and organic acids content. VOCs production is directly related to the ripening behavior. Climacteric melons tend to produce more esters, giving fruity aroma, whereas non-climacteric melons contain a greater percentage of aldehydes, having cucumber-like flavor [1, 2]. The relationship between aroma and ripening has been extensively studied. Ethylene, the hormone regulating the onset of ripening in climacteric cultivars, induces the expression of several genes involved in volatile biosynthesis, such as branched-chain amino acid aminotransferase (*CmBCAT1*), aromatic amino acid aminotransferase (*CmArAT1*), alcohol acyltransferases (*AAT*s), and alcohol dehydrogenases (*ADH*s) [3–6]. However, knowledge about the genetic regulation of aroma production in melon is still scarce [7].

To decipher the genetic control of agronomic traits in melon, mapping populations have been developed, such as Recombinant Inbred Lines (RILs) and Introgression Lines (ILs). ILs are useful tools for Quantitative Trait Loci (QTL) identification, as well as for fine mapping and QTL interaction studies. In melon, several IL collections have been developed using genetically distant parental accessions, such as PI 161375 *(ssp. agrestis*, *chinensis* group) in the ‘Piel de Sapo’ (PS) (*ssp. melo*, *inodorus* group) background [8] and PI 420176 (*ssp. agrestis*, *makuwa* group) in the ‘Védrantais’ (VED) background (*ssp. melo*, *cantalupensis* group) [9]. Recently, two reciprocal IL collections have been developed between the commercial varieties PS and VED [10, 11]. In addition, among several RIL populations developed in melon [12–14], the volatile profile has been evaluated in a VED x PS RIL population, and 166 VOCs QTLs have been detected [2].

The aim of this work is to analyse the volatile profile of the two reciprocal VED x PS IL populations to validate the QTLs previously detected in the VED x PS RIL population and to detect new QTLs. Moreover, we want to narrow down the genomic interval of some interesting QTLs for VOCs production to identify the underlying candidate genes. This knowledge will help to identify genes controlling ripening and volatile biosynthetic pathways, which will be useful for the improvement of melon flavor.

## Results

### Fruit quality QTLs in the IL collections

ILs were phenotyped for fruit quality (fruit weight (FW), length (FW), width (FWI), perimeter (FP), area (FA) and solid soluble content (SSC)) and ripening traits (flesh firmness (FIR), earliness of yellowing (EYELL), earliness of chlorophyl degradation (ECD), earliness of aroma production (EARO), earliness of abscission layer activation (EALF), abscission level (ABS) and harvest date (HAR)) (Supplementary Table S1). In the PS ILs, PS2.2, PS6.1 and PS7.1_C showed bigger fruits compared to the recurrent parent VED, affecting FW, FL, FWI, FA and FP, while line PS10.2_C produced smaller fruits. A firmness QTL was identified in PS3.3, having harder flesh. Regarding ripening traits, line PS6.1 showed a two-day delay in EARO and ECD. Line PS10.2_C also presented a 2 to 3-day delayed ripening phenotype, affecting EALF, ECD, HAR and ABS.

In the VED ILs, line VED6.4_C showed a 35% increase in FW, whereas as 38% reduction was observed in VED7.1_C and VED7.2_C. Regarding fruit morphology, line VED5.1_C presented increased FW and FL, while VED8.2 and VED11.2_C produced more spherical fruits. Two lines showed SSC QTLs affecting sugar accumulation in opposite directions. Line VED7.1_C showed a 1.6°Bx increase, whereas VED10.2 melons presented 2.9°Bx less than PS. Firmness was increased in line VED11.2_C. Regarding fruit ripening, EYELL QTLs were identified in lines VED3.4, VED8.3_C and VED10.2, showing earlier, delayed and no yellowing phenotypes, respectively. An EALF QTL was identified in line VED11.2, showing a lignified star-shaped layer surrounding the fruit pedicel (Supplementary Fig. S1). Finally, early ripening phenotypes were observed for lines VED7.1_C, VED8.2 and VED8.3_C, showing QTLs for HAR, EALF and EARO.

### General aroma profile in the IL collections

**Figure 1:**
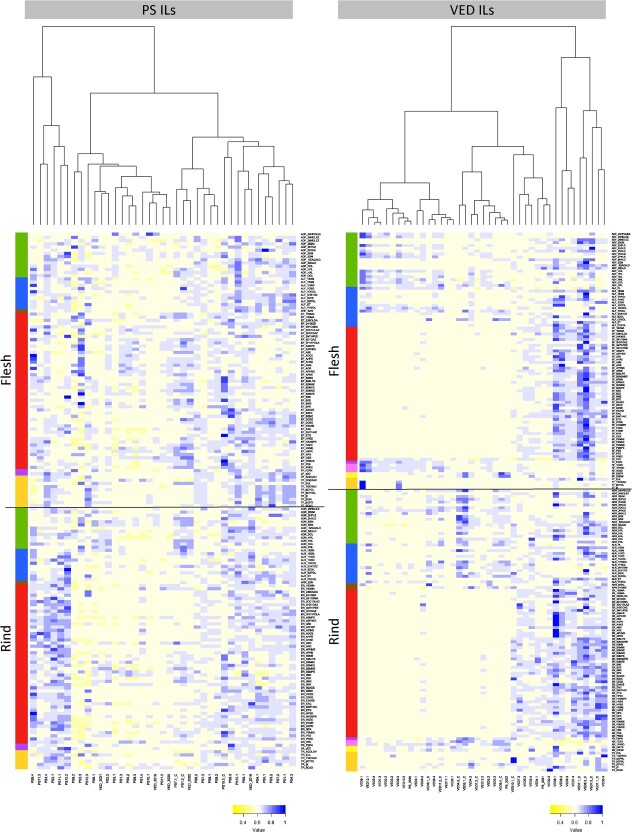
Heatmap showing the correlation matrix between ILs and volatile compounds, and ILs dendrograms for both PS ILs and VED ILs populations. Chemical groups are coded on the left ordered from top to bottom: aldehydes (green), alcohols (blue), aromatic/benzenoids (brown), esters (red), furans and lactones (purple), ketones (pink), sulphurs (yellow) and terpenes (orange).

In this work, 160 different VOCs were detected, 91 of which were observed in all harvest seasons (Supplementary Tables S2, S3 and S4). PS ILs presented scattered variation within the heatmap, not appreciating big differences overall. However, PS2.1, PS4.4, PS9.4, PS11.1, PS11.3 and PS12.2 showed a different profile in rind compared to the rest of the lines, correlating more than the average with some esters and alcohols. On the other hand, lines PS3.3 and PS8.2 presented the lowest correlation values with esters in rind. Interestingly, PS3.3 had an opposite behavior in flesh, correlating with several esters (Fig. 1). VED ILs formed a two-block dendrogram. Most of the lines clustered with the PS parental line. However, 9 lines showed great differences in their aromatic content, having high ester production: VED1.1_C, VED1.4, VED5.4, VED6.3, VED7.1_C, VED7.2_C, VED8.1, VED8.2 and VED8.3_C. Statistical analyses revealed a total of 263 and 983 QTLs in the PS IL (Supplementary Table S5a) and the VED IL (Supplementary Table S5b) collections, respectively.

### Volatile QTLs in the PS ILs

#### QTLs reducing ester production in VED background

**Figure 2:**
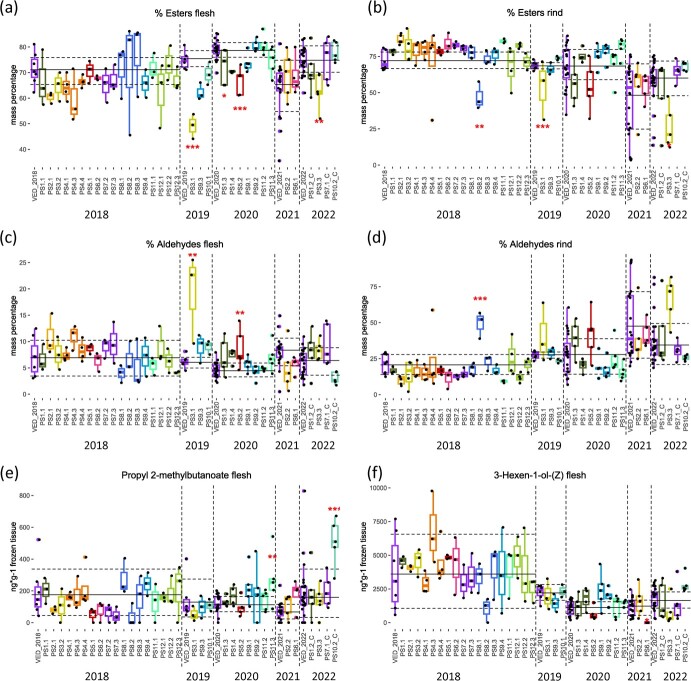
Boxplots showing differences in VOCs levels in PS ILs collection. Each dot represents a replicate, the solid line corresponds to the average and the dashed lines to the average ± SD of the recurrent parent VED. Asterisks represent *p*-values (* <0.05; ** <0.01; *** <0.001).

The aldehyde-ester balance was affected in favor of aldehydes in PS3.1, PS3.3, PS5.2 and PS8.2 in both flesh and rind (Fig. 2a-d). QTLs for specific VOCs were also identified in this ILs (Supplementary Table S5, Fig. 2e-f).

Lines PS3.1 and PS3.3 showed an important decrease in ethyl ester content in both tissues. In PS3.1 acetate content was also reduced in rind and aldehyde abundance was increased from 6% to 11–19% in flesh. On the other hand, butyl, propyl and phenyl esters were increased. Aliphatic and phenolic aldehydes were also accumulated, mainly in flesh tissues.

In line PS5.2, the aldehyde-ester balance was only affected in flesh, reducing esters from 79% to 67% and almost doubling the aldehyde percentage of VED melons.

PS8.2 effects were mainly found in rind. An increased aldehyde proportion almost reaching 50% and a 90% reduction in ethyl acetate and ethyl hexanoate were observed.

Lower ester content was found in other lines. Line PS4.4 had an 80% lower content in ethyl acetate and ethyl propanoate compared to VED. Line PS6.1 production of 3-hexen-1-ol acetate-(Z) was 95% decreased compared to VED, probably related to the null detection of the precursor 3-hexen-1-ol-(Z) (Fig. 2f). The absence of these VOCs in PS6.1 made this QTL a suitable candidate for a fine mapping strategy.

#### QTLs increasing ester content in VED background

The climacteric profile of VED fruits, characterized by a high ester content, was enhanced in several PS ILs. Interestingly, the 6 lines grouping apart in PS ILs did not present a great number of ester QTLs in rind, except for PS11.3, making this clustering less explanatory than in VED ILs. Lines PS2.1 and PS2.2 showed up to a 300% increase in branched-chain amino-acid-derived esters and some acetates (Supplementary Table S5a). In line PS6.1, an increased content in butyl, butanoate and sulphur esters was observed. This phenotype was coupled to an increase in C9 aldehydes, 1-heptanol and 1-hexanol. Three esters were found to be overproduced in PS7.1 and PS7.1_C compared to VED. PS ILs in chromosome 9 were found to produce more aliphatic and branched esters. However, line PS9.3, containing the overlapping region between PS9.2 and PS9.4, had a reduced content in three ethyl esters. Ester content was also increased in lines PS10.1, PS10.2 and PS10.2_C. Finally, several ester QTLs were found in PS11.3, including 2-methylbutanoate derivatives, ethyl benzeneacetate and two acetates (Fig. 2e). Line PS11.3 was selected to fine map the genomic interval to unveil the gene responsible for ester increase.

#### Terpenoid QTLs

Terpenoids include several volatile compounds, such as apocarotenoids, monoterpenes and sesquiterpenes. Several regions containing terpenoid QTLs enhanced by PS alleles in VED background were found (Supplementary Table S5a). For instance, 2 QTLs for cis-geranylacetone were identified in PS1.1, PS1.3 and PS1.4. On chromosome 2, PS2.1 showed an increased content in trans-geranylacetone and β-cyclocitral in rind. An increase in apocarotenoid content was also observed in PS4.1 and PS4.4 in flesh. Terpineol and eucalyptol were highly produced in PS6.1. Moreover, many apocarotenoid QTLs were detected in PS10.1 flesh, and a β-cyclocitral QTL was identified in PS12.3. On the other hand, QTLs for reduced terpenoid content were identified in PS5.2, PS8.2 and PS11.3, affecting eucalyptol.

### Volatile QTLs in the VED ILs

#### Ester-producing lines

**Figure 3:**
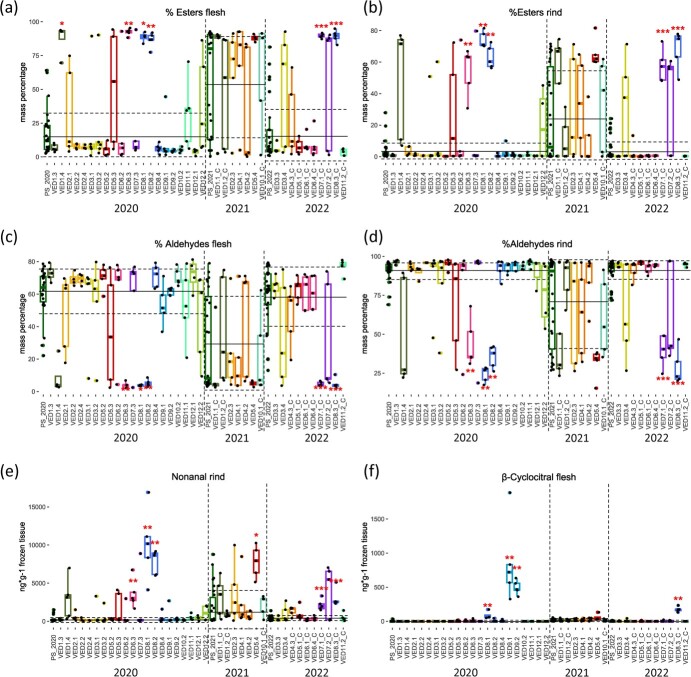
Boxplots showing differences in VOCs levels in VED ILs collection. Each dot represents a replicate, the solid line corresponds to the average and the dashed lines to the average ± SD of the recurrent parent PS. Asterisks represent *p*-values (* <0.05; ** <0.01; *** <0.001).

Lines forming a separated cluster in the VED ILs dendrogram contained a high number of ester QTLs, except for VED1.1_C. In VED1.1_C, differences in esters were only found for pentyl acetate. High increases in esters percentage and reductions in aldehyde proportion were observed in lines VED6.3, VED7.1_C, VED8.1, VED8.2 and VED8.3_C (Fig. 3a-d), detecting stronger changes than in PS ILs. Interestingly, these lines also presented greater quantities of C9 aldehydes such as nonanal, C8 alcohols such as 1-octanol, and other volatiles such as 2-methylbutanol and eucalyptol (Supplementary Table S5b) (Fig. 3e). Milder phenotypes were observed in VED1.4, VED5.4 and VED7.2_C, also showing a high ester production, but without affecting aldehyde percentage (Supplementary Table S5b).

QTLs for phenylalanine-derived esters were found in VED10.2 and VED11.2_C. A QTL for methyl benzoate was detected in two years in VED10.2. On the other hand, an ethyl cinnamate QTL was only identified in 2 out of 5 replicates in VED11.2_C.

#### Apocarotenoid QTLs

The orange-fleshed lines VED9.1 and VED9.2 contained QTLs for apocarotenoids such as β-cyclocitral, β-ionone epoxide and trans-geranylacetone (Fig. 3f). A small production of apocarotenoids was also detected in VED8.1, VED8.2 and VED8.3_C (Supplementary Table S5, Fig. 3f). Finally, many ILs showed increased abundances of eucalyptol such as VED5.4, VED6.3, VED7.1_C, VED8.1, VED8.2 and VED8.3_C.

### Fine mapping of a lipid-derived QTL cluster on chromosome 6

**Figure 4:**
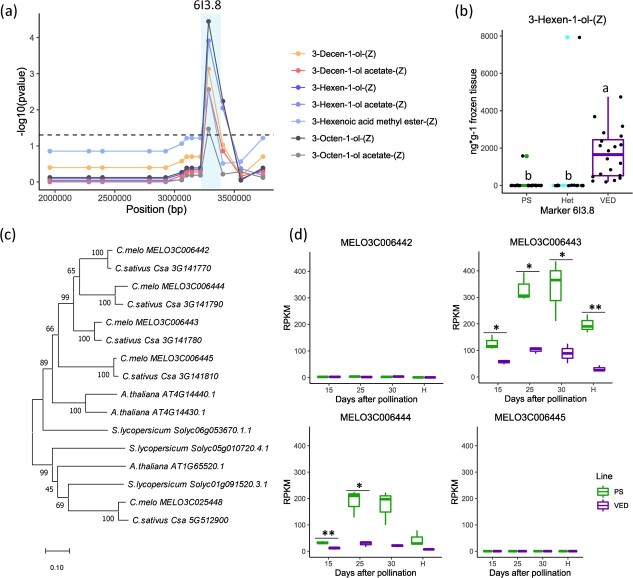
Fine mapping of a lipid-derived QTL cluster on chromosome 6. (a) Association by Wilcoxon test between VOCs production and molecular markers for F3 recombinant plants. (b) Production of 3-hexen-1-ol-(Z) in F2 recombinant lines, comparing PS, heterozygous (Het) or VED alleles for marker 6I3.8. Lower case letters indicate significant differences (*p* < 0.05, Wilcoxon test). (c) Phylogenetic tree of enoyl-CoA isomerases from melon, cucumber, *Arabidopsis* and tomato. (d) Candidate genes expression obtained by RNA-Seq in fruit flesh at different ripening stages (n = 3). Asterisks represent the *p*-values (* <0.05; ** <0.01; *** <0.001).

The absence of 3-hexen-1-ol-(Z) and 3-octen-1-ol-(Z) and a reduction in 3-hexen-1-ol acetate-(Z) were observed in PS6.1 (Supplementary Table S5a). This phenotype was related to C6 QTL cluster found by Mayobre et al. (2021), delimited to a 1.78-Mb region containing 267 genes [2]. Among the differentially produced VOCs between sub-IL SUB-PS6.1a and VED melons, 3-hexen-1-ol-(Z), 3-hexen-1-ol acetate-(Z), 3-octen-1-ol-(Z), 3-octen-1-ol acetate-(Z), 3-decen-1-ol acetate-(Z) and methyl 3-hexenoate-(Z) were associated with the original QTL. Analysis of F2 and F3 recombinant lines revealed a significant association with marker 6I3.8 (Fig. 4a). The PS allele was shown to be dominant, as heterozygous lines behaved similarly (Fig. 4b). The final 188-Kb genomic interval, between markers 6I3.6 and 6I4, contained 30 annotated genes (Supplementary Table S6). Among these genes, a cluster of 4 enoyl-CoA isomerase 2 (ECI2) genes was found (*MELO3C006442, MELO3C006443, MELO3C006444 and MELO3C006445*). An ortholog search in PLAZA and a phylogenetic analysis revealed their similarity to delta3-delta2 enoyl-CoA isomerases 2 and 3 from *Arabidopsis thaliana* (Fig. 4c). RNA-Seq data confirmed that only *MELO3C006443* and *MELO3C006444* were expressed in melon fruit flesh, both genes showing higher expression in PS than in VED during fruit ripening (Fig. 4d). A few mutations were identified in the coding sequence of both genes between PS and VED, however none of them was predicted to be deleterious according to Provean (https://www.jcvi.org/research/provean) and SIFT tools (https://sift.bii.a-star.edu.sg/) (Supplementary Fig. S2).

### Fine mapping of an ester production QTL on chromosome 11

**Figure 5:**
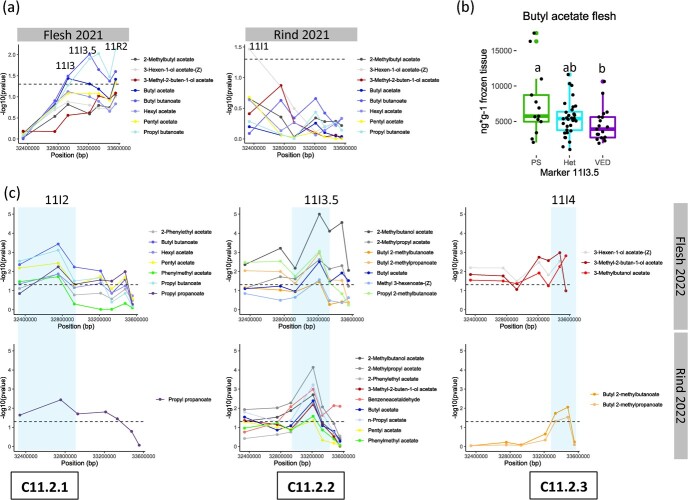
Fine mapping of ester production QTL on chromosome 11. (a) Association by Wilcoxon test between VOCs production and molecular markers for F2 recombinant plants. (b) Relative amount of butyl acetate found in F2 recombinant lines, comparing PS, heterozygous (Het) or VED alleles for marker 11I3.5. Lower case letters indicate significant differences (*p* < 0.05, Wilcoxon test). (c) Association by Wilcoxon test between VOCs production and molecular markers for F3 recombinant plants in three significant regions found.

The distal part of chromosome 11 had a QTL related to ester abundance in the VED background, previously detected in a RIL population [2] and validated in line PS11.3. Twenty compounds differentially produced between sub-IL 11PS and VED were selected for the analysis, including some esters in the original QTL [2] (Supplementary Table S2). The QTL interval was delimited to 1.97 Mb containing 234 genes. F2 and F3 recombinant lines generated from the PS11.3 x VED cross were analysed. Data from F2 recombinants showed a significant association in flesh from marker 11I3 to 11R2 (Fig. 5a). Significant differences were found between homozygous individuals (*p*-value = 0.049), but not with heterozygous, as these plants showed an intermediate phenotype (Fig. 5b). Interestingly, progeny test F3 lines suggested the existence of three significant associated regions in both tissues, flesh and rind, at the beginning, in the middle and at the end of the QTL interval (Fig. 5c).

## Discussion

### QTLs for fruit quality

ILs phenotyping contributed to confirm previously described QTLs and to identify new QTLs associated with fruit quality and ripening.

In the PS ILs in VED background, a QTL for fruit size in PS2.2 showed an opposite effect to the one detected in 2020 [10]. This difference could be attributed to environmental conditions. QTLs for FW and FS were also identified in the same genomic region of chromosome 2 in other melon populations [14–16], indicating this region contains a regulator of fruit morphology. Additionally, the FW QTL located in PS6.1 colocalizes with a previously described QTL in a PS x PI 161375 IL population [17]. Regarding bigger fruits of PS7.1_C, this phenotype was not previously detected in PS7.1, likely due to the presence of non-targeted introgressions that were removed in PS7.1_C [10]. This QTL colocalizes with *FWQW7.1* in the VED ILs population [11] and with a fruit size QTL described in a PI 414723 x Dulce RIL population [12]. The smaller fruits of PS10.2_C could be related to *FAQP10.1* and *FPQP10.1*, QTLs previously identified in PS10.1 [10], but also to a FW QTL detected by Eduardo et al. (2007) [17]. The differences in size with PS10.2 may be due to PS10.2 having a non-targeted introgression on chromosome 2, shared with the elongated line PS2.1 [10]. The delay in ripening observed in PS6.1 could be related to *EAROQP6.1*, previously identified in line PS6.2 [10] and explained by the presence of *CmNAC-NOR* (*MELO3C016540*) (Supplementary Fig. S3), a central regulator of ripening in melon [18]. New QTLs associated with delayed ripening were identified in PS10.2_C, which can contribute to the development of long shelf life varieties.

In the VED ILs in PS background, SSC QTLs were newly identified in lines VED7.1_C and VED10.2, which could be used to modulate sugar content in commercial varieties. SSC accumulation is complex, dependent on different sugars and highly affected by environmental conditions [19]. These SSC QTLs were only found in a single experiment, so these lines should be tested again. On the contrary, the firmness QTL detected in VED11.2_C was stable, as it was previously detected in VED11.2 [11]. Fruit size QTLs detected in this population were also coincident with previously described QTLs. For instance, the VED6.4 introgression, increasing size, overlaps with a region described by Perpiñá et al. (2016) [9]. The small fruits observed in VED7.1_C and VED7.2_C, validating VED7.1 and VED7.2 phenotypes [11], colocalize with *FPQW7.1* (Supplementary Table S1) and another QTL described by Harel-Beja et al. (2010) [12]. FS QTLs on VED5.1_C, VED8.2 and VED11.2_C also collocated with previously described QTLs [9, 20]. EYELL QTLs found in VED8.3_C and VED10.2 (containing *CmKFB* [21]) confirmed the previous phenotyping of the population [11], and a new region in line VED3.4 was found. The genes affecting the earliness of this phenotype on chromosomes 8 and 3 are still unknown. A QTL for abscission was previously reported in lines PS11.1 and PS11.3 [10], an effect that had not been observed in VED ILs reciprocal lines [11]. Interestingly, the activation of the abscission layer was observed in most melons from line VED11.2_C around 36 Days After Pollination (DAP) (Supplementary Fig. S1), never resulting in full slip. VED11.2 melons did not exhibit other ripening traits such as aroma production, nor senescence, being collected at 55–56 DAP as the rest of non-climacteric fruits. A potential value of the abscission phenotype found in line VED11.2_C for breeding non-climacteric melons remains to be determined. Three ripening-associated QTLs for EARO, EALF and HAR were confirmed in 3 different regions on chromosome 8. Line VED8.3_C maintained the climacteric behavior after removal of an overlapping SNP with VED8.1 [11]. The climacteric behavior presented in line VED7.1 was also preserved in VED7.1_C after removal of the chromosome 8 introgression. This phenotype might be related to ripening-related QTLs identified in a VED x PS RIL population [22], making it an interesting region for further studies.

### General aroma profile in the IL collections

When analysing the general correlation among the introgression lines, a different pattern based on the genetic background was observed. Specifically, it was noted that a small number of individual key introgressions can greatly impact the aromatic profile in the non-climacteric PS, whereas in a climacteric VED background, a larger number of genomic regions can make small measurable changes in the volatile content. A positive correlation between climacteric VED ILs and ester production was observed. The total number of volatile QTLs identified in this study was greater than in other GC–MS studies using different melon IL populations [23, 24], providing the basis for the elucidation of the full volatile biosynthesis pathways and for breeding varieties with improved flavor. These differences could be attributed to the higher number of volatiles measured in our study. The nature of these QTLs is further discussed in the subsequent sections.

### Volatile QTLs in the PS ILs

#### QTLs reducing ester production

Esters and aldehydes constitute the main components of the melon volatile profile, comprising approximately 90% of the VOCs content [2]. Esters and aldehydes abundance is usually balanced, as aldehydes can be transformed into esters. Ethylene triggers this step by activating the expression of alcohol dehydrogenase (ADH) and alcohol acyltransferase (AAT) genes [3, 5]. Therefore, changes in climacteric behavior can affect ester and aldehyde proportions.

The reduction in esters and the increase in aldehydes observed in line PS8.2 may be related to the presence of the ripening QTL *ETHQV8.1*, playing an important role in ethylene production, abscission, aroma and harvest date [11, 22, 25]. Additionally, QTLs for ethyl hexanoate, ethyl acetate and benzeneacetaldehyde were identified in previous studies in the same region [2, 26].

Regarding line PS3.1, also presenting a ‘less climacteric’ volatile profile, QTLs for acetates had been identified in the same genomic region [26]. However, our observations reveal an increase in aldehydes that cannot be explained by these QTLs. To our knowledge, this is the first time that this segment of chromosome 3 is associated with such volatile changes in melon. Within the PS3.3 region, QTLs for aroma production have been previously reported in the VED x PS RIL population [22]. Moreover, QTLs for sulphur esters *E_EMTHAC_P3.3* and *E_MMTHAC_P3.3* match the same region for *EF_EMTHAC_3.1* [2]. An ethylene-related gene or the presence of *CmBCAT1* (*MELO3C010776*) in this interval, differentially expressed between VED and PS (Supplementary Fig. S3) and reported to be responsible for the deamination of branched amino acids [4], may be associated to some of these QTLs. Several QTLs identified in PS5.2 correspond to previously identified regions in the VED x PS RIL population. For instance, *E_PNEE_P5.2* matches *EF_BEE_5.1*, another QTL for ethyl esters [2]. An increase in n-propyl acetate content coincides with QTL *EF_NPA_5.1*, while the higher levels of C9 aldehydes could also be related to *ADF_NNL_5.1*, *ADF_NNL_5.2* and *ADF_NNL_5.3* QTLs [2].

We suggest that chromosomes 3, 5 and 8 play an important role in the ester-aldehyde balance. Some candidate genes have been proposed for *ETHQV8.1* [22], but additional efforts are needed to identify the genes underlying the QTLs on chromosome 3 and 5, which would be of great interest for breeding flavor in cantaloupes.

Regarding minor esters changes, *E_EAC_P4.3* and *E_PEE_P4.3* QTLs on chromosome 4 enclose the *CmPDC1* gene (*MELO3C009145*), responsible for the decarboxylation of α-keto acids to form straight chain aldehydes [27]. These aldehydes are precursors of ethyl, propyl, and pentyl esters. Thus, this gene could be the candidate for the two identified QTLs as it is differentially expressed between VED and PS at harvest (Supplementary Fig. S3).

Interestingly, we did not detect ester QTLs containing the well-known genes *CmAAT1*, *CmAAT3* and *CmAAT4* on PS ILs, despite the fact that a QTL cluster on the *CmAAT1–3* region had been described in the VED x PS RIL population [2]. The absence of ester QTLs in this region on the ILs could be explained by the low effect of the QTL previously described, or the need of AATs to interact with other QTLs not present in the ILs but present in the RILs.

#### QTLs increasing ester content

The introgression of non-climacteric PS alleles into a climacteric VED background resulted in some lines with increased ester content, as previously seen by Mayobre et al. (2021) [2]. Regarding ester QTLs in line PS2.1, they might be associated to previously described ripening or isoleucine-derived ester QTLs [2, 22]. Isoleucine-derived volatiles such as 2-methylbutanoate esters were suggested as products of threonine metabolism [28]. In fact, threonine aldolase *CmTHA1* (*MELO3C010162*) is located within PS2.1 and P2.2 introgression lines, an association with *E_B2M.PE_P2.1* and *E_B2MME_P2.2* QTLs being possible (Supplementary Fig. S3).

Several ester QTLs identified on line PS6.1 could be related to a sulphur ester QTL previously mapped on chromosome 6 [26]. However, this match cannot explain other ester QTLs in the region.

The effects behind PS7.1 and PS7.1_C increase in acetate-containing volatiles might be related to primary metabolism. This suggestion is based on a recent study that links ester formation to changes in the expression of primary metabolism enzymes such as pyruvate and malate dehydrogenases or long-chain acetyl-CoA synthetases [29].

Several ester QTLs were detected on chromosome 9 ILs, sharing a 14.9-Mb region of the chromosome. The differences between overlapping lines may be due to a different harvest year, the presence of non-detected small introgressions in some lines, or a polygenic regulation on chromosome 9 affecting esters.

Esters were increased in lines PS10.2 and PS10.2_C. The larger number of QTLs observed in PS10.2_C may be due to the larger number of replicates analysed or to the existence of a small introgression on chromosome 2 in PS10.2. The high number of ethyl ester QTLs in PS10.2_C could be related to the delayed ripening of the line, as the fruit takes longer to ripe and can result in a higher VOCs accumulation.

The region covered by PS11.3, presenting enhanced ester content, was coincident with ester QTLs described in Mayobre et al. (2021) [2] and a fine mapping strategy was started in this work.

#### Candidate genes in terpenoid production

VED is an orange-fleshed melon, its color mainly conferred by carotenoid accumulation. However, PS alleles demonstrated to be able to promote the generation of volatiles derived from these compounds. The great number of QTLs identified for terpenes and apocarotenoids highlights the polygenic regulation of the metabolic pathways involved. In addition, the effect on terpene accumulation was not observed to correlate with ripening QTLs. Although most QTLs were detected in flesh, some QTLs were detected in rind, suggesting a differential regulation between both tissues. QTLs on chromosomes 2 and 6 are an example of this variation. Interestingly, no change in terpenes was found in the green-fleshed PS9.4, suggesting apocarotenoid production from carotenoids is possible in similar quantities to VED.

Several previously identified QTLs were found to be coincident with our QTL regions. Some sesquiterpene QTLs detected in PS6.1 were identified in the same region [26]. This was also the case for PS10.1, as QTLs for β-carotene were found within this region [26].

Carotenoid cleavage dioxygenases (CCDs) are key steps in the apocarotenoid formation in melon. Indeed, the region of QTL *T_CGERAC_P1.3* contains *MELO3C023555*, also known as *CmCCD1*, involved in geranylacetone, pseudoionone, α-ionone and β-ionone biosynthesis [30] and expressed in VED and PS flesh (Supplementary Fig. S3). Other CCDs are annotated in the melon genome, having a high degree of sequence similarity with *CmCCD1*, and could be related to other apocarotenoid QTLs, although not all of them are expressed in fruit tissue.

Carotenoid isomerases might also influence volatile formation. For instance, *MELO3C009571*, showing differential expression in VED and PS flesh, may explain apocarotenoid QTLs in line PS4.4 (Supplementary Fig. S3).

More studies should be done to demonstrate the relationships stated with the putative candidate genes. As terpenoids have a great diversity of aromatic notes, from floral to minty, it could be of great interest to uncover the genes underlying these regions.

### QTLs for volatiles in the VED ILs

#### The effect of climacteric ripening in aroma

In contrast to the non-climacteric recurrent parent PS, climacteric behavior was observed in lines VED6.3, VED7.1, VED8.1, VED8.2 and VED8.3_C [11] in agreement with their volatile profile observed in this work. Many previous studies have underlined the tight relationship between the ripening behavior and the volatile profile, being esters more abundant in climacteric melons [1, 31]. The abundance in C8 and C9 alcohols and aldehydes could be related with maturity, as C8 and C9 VOCs have been previously linked to ripeness [32]. These volatiles could be treated as biomarkers of maturity. The aroma of these lines seems to be a consequence of the presence of genes related to climacteric ripening. For instance, the knock-out of *CmNAC-NOR*, a gene covered by line VED6.3, prevented ripening, with no ethylene production and a big decrease in ester content [18, 33]. VED6.3 did not show an enhanced content in C8 and C9 aldehydes, suggesting that *CmNAC-NOR* is exclusively related to ester formation. The climacteric behavior observed in line VED7.1_C was coupled to an increase in esters. VED7.1_C had overlapping volatile QTLs with VED7.2_C, but VED7.2_C presented an unstable behavior, 3 replicates being climacteric and 2 being non-climacteric. This region matches previously described ripening QTLs [22]. Lines VED8.1, VED8.2 and VED8.3_C shared many QTLs but also had some unique QTLs. These lines are thought to represent 3 different QTLs affecting climacteric ripening and aroma, as VED8.2 is the only one carrying the validated QTL *ETHQV8.1* [22], having effects in aroma production [25], and the other two lines present a divergent behavior in abscission [11]. The phenotype of line VED8.3_C, which does not overlap with VED8.1, supports this hypothesis. The VED8.1 phenotype was tentatively linked to the earliness of flowering QTL *DtF8.1*[13] by Santo Domingo et al. (2022) [11]. Many QTLs had been previously mapped to chromosome 8 [2, 26], also having a reciprocal effect in both PS ILs and VED ILs populations, highlighting the importance of this region in the regulation of volatile pathways.

Looking into non-climacteric lines, the increased ester content of VED3.4 could be related to *AROQV3.1*, identified in the VED x PS RIL population [22], or to ripening QTL *ETHQB3.5*, described in another population and containing a NAC transcription factor [34, 35]. Moreover, some branched-chain compounds might be related to the presence of *CmBCAT1* (*MELO3C010776*), involved in amino acid deamination [4]. Reciprocity with the PS3.3 line was only observed for ethyl benzoate and 2-methylbutyl 2-methylpropanoate. The observation of ester QTLs in VED1.4, VED3.4 and VED5.4, could be due to the presence of unstable non-detected minor QTLs related to ripening. In addition, the fact that line VED1.1_C clusters with climacteric lines but only contains one ester QTL suggests our statistical method might be too restrictive to detect small differences in volatile content, or that the high variability found in some replicates could partially affect significancy.

A methyl benzoate QTL was observed in the non-climacteric line VED10.2, suggesting an ethylene-independent synthesis of this compound. Methyl benzoate has a prune, floral, sweet flavor, although it was detected below the reported odor threshold. The biosynthesis pathway of this VOC is partially known, coming from the methylation of benzoic acid by *CmBAMT* (*MELO3C003803*) [36]. However, this enzyme is located on chromosome 4. A 3′-N-debenzoyl-2′-deoxytaxol N-benzoyltransferase (*MELO3C011816*) is annotated on the VED10.2 interval. Expression data highlights *MELO3C011816* as a good candidate gene, putatively related with the availability of benzoyl-CoA (Supplementary Fig. S3). This QTL is of great interest due to the stability and reproducibility of the phenotype, observed in two different years. In addition, two phenylpropanoid-related genes expressed in fruit flesh, *CmPAL13* (*MELO3C025786*) and *CmC4H1* (*MELO3C019585*), were found within the VED11.2 interval, which could be related with an ethyl cinnamate QTL (Supplementary Fig. S3).

#### *CmOr* and minor QTLs effect on terpenoids accumulation

Orange-fleshed VED ILs, VED9.1 and VED9.2, are the ones producing higher amounts of apocarotenoids, suggesting that the *CmOr* gene, responsible for the orange flesh, is the causal gene [37] (Supplementary Fig. S3). PS might have the genetic machinery to cleave carotenoids, as apocarotenoids appear when *CmOr* is introgressed in the PS background. The identification of minor QTLs on chromosome 8 was remarkable. On one hand, VED8.3_C QTL could be related to the green flesh phenotype, whereas for VED8.1 and VED8.2 further analyses should be performed on the carotenoid content to explain the presence of β-cyclocitral.

Eucalyptol QTLs were identified in ester-producing lines such as VED5.4, VED6.3, VED7.1_C, VED8.1, VED8.2 and VED8.3_C, probably implying a ripening-associated regulation.

### The link between β-oxidation and lipid-derived volatiles on chromosome 6

The strong reduction in lipid-derived volatiles observed in line PS6.1 matched C6 cluster for lipid-derived volatiles previously identified by Mayobre et al. (2021) [2]. 3-Hexen-1-ol-(Z) and 3-octen-1-ol-(Z) have green flavor, but their respective acetates are described as fruity, therefore it was interesting to decipher the gene underlying this QTL. Moreover, the amounts detected for 3-hexen-1-ol-(Z) and 3-hexen-1-ol acetate-(Z) in VED melons were above their reported odor thresholds, suggesting that a reduction in these volatiles in PS6.1 could be detected by consumers. The fine mapping of the region allowed the selection of 2 enoyl CoA isomerases (ECI), *MELO3C006443* and *MELO3C006444*, as putative candidate genes. A recent transcriptomic study linked 3-hexen-1-ol-(Z) and the corresponding acetate to 3Z-2E enoyl-CoA isomerases [32]. Nevertheless, the gene code given in the study was not included in our interval. ECIs are known to be involved in the β-oxidation pathway, transforming 3Z double bonds into 2E double bonds and facilitating the degradation of fatty acids. The higher expression of these enzymes in PS compared to VED matches the reduction in 3Z VOCs in the PS6.1 introgression line. In Arabidopsis, 3 ECIs have been identified and studied [38]. Genic duplications of these enzymes are known to have happened along the evolution of both Arabidopsis and melon according to PhylomeDB (http://phylomedb.org/). Therefore, both ECIs could be responsible for the observed phenotype in PS6.1. Comparing the sequences to their Arabidopsis orthologs, the peroxisomal C-terminal signal PTS1 (-SKL) and the glutamate catalytic residue were observed in both genes for both PS and VED alleles [38, 39]. Future studies will help to elucidate whether both enzymes are active for the same substrates.

### The complex control of ester production by chromosome 11

Line PS11.3 was found to produce more esters than VED, this phenotype being associated to C11.2 cluster in Mayobre et al. (2021) [2]. Fine mapping revealed at least three different sub-QTLs. C11.2.1, from 32 338 980 to 32 928 874 bp, is associated to aliphatic and aromatic acetates, and butanoate and propanoate derivatives. Within the 65 genes included in the interval, pyruvate dehydrogenase E1 component subunit alpha (*MELO3C022306*) is one of the most suitable candidates (Supplementary Table S7)(Supplementary Fig. S3). The levels of expression of this enzyme were recently related to the ester abundance, as pyruvate can be transformed to acetyl-CoA, a precursor of acetates [29]. Another candidate in this region could be a MADS-box transcription factor (TF) (*MELO3C022316*) (Supplementary Fig. S3). MADS-box TFs have been related to the ethylene signaling pathway in tomato, although in melon NAC TFs are thought to play a more relevant role [40]. C11.2.2, from 32 928 874 to 33 332 519 bp, is the highest associated region to most of the esters analysed. This region contains 57 annotated genes. Pyruvate dehydrogenase E1 component subunit beta (*MELO3C022343*) and succinate-CoA ligase [ADP-forming] subunit beta (*MELO3C022382*) could be involved in ester formation, but also ethylene-responsive transcription factor ERF113-like (*MELO3C022358*), as ethylene is known to trigger ADHs and AATs [3, 5]. C11.2.3, from 33 332 519 to 33 555 451 bp, containing 27 annotated genes, could be associated with an ADH-like gene (*MELO3C022399*). All these enzymes are expressed in fruit flesh during ripening (Supplementary Fig. S3). In addition, some modifier variants were found in *MELO3C022316* and *MELO3C022343* genes (Supplementary Table S7). However, more studies are needed to decipher the genes underlying the observed changes in aroma, which could be applied in fruit breeding to enhance fruity flavor.

## Materials and methods

### Plant material

Two reciprocal IL populations were evaluated: 33 PS ILs (PS introgressions in VED) and 36 VED ILs (VED introgressions in PS), generated from a cross between the non-aromatic, non-climacteric ‘*Piel de Sapo*’ T111 (PS) (*Cucumis melo ssp. melo, inodorus* group) and the aromatic, climacteric ‘*Védrantais*’ (VED) (*C. melo ssp. melo, cantalupensis* group) [10, 11]. Fifteen ILs were cleaned of undesired introgressions by backcrossing them with their recurrent parent (VED or PS): PS1.2, PS7.1, PS10.2, VED1.1, VED1.2, VED4.3, VED5.1, VED6.1, VED6.4, VED7.1, VED7.2, VED8.3, VED8.4, VED10.1 and VED11.2. After self-pollination to fix the target introgression, these ILs were renamed with a ‘_C’. In addition, three new lines were generated covering missing regions in the original IL sets, using pre-ILs as starting point: PS3.3, VED3.3 and VED3.4 (Supplementary Table S8). Plants were grown during summer seasons from 2018 to 2022 under greenhouse conditions at Caldes de Montbui (Barcelona), allowing only one fruit per plant. Due to the interesting phenotype of lines PS3.1, PS5.2, VED6.2, VED8.2 and VED10.2, they were evaluated in two different years (Supplementary Table S8). Fully ripe fruits were harvested as previously described [22]. PS ILs fruits were mostly harvested in 2018, and some lines between 2019 and 2022. VED ILs fruits were mostly harvested in 2020, and some lines in 2021 and 2022. Three to five fruits of each IL and 8 to 40 fruits for PS and VED parental lines were sampled for the volatile analysis in all the trials performed from 2018 to 2022.

Two fine mapping populations were generated for QTL clusters C6.1 and C11.2, described in Mayobre et al. (2021) [2] (Supplementary Table S9). For C6.1, P61_28, a pre-IL presenting heterozygosis on chromosome 6 in VED genetic background, was chosen for self-pollination and selection of recombinants between QTL flanking markers (Supplementary Table S9a). Moreover, a sub-IL called SUB-PS6.1a, containing a smaller introgression covering the QTL, was also generated. Forty-nine recombinant F2 plants, 4 SUB-PS6.1a and 6 VED were sown and self-pollinated in 2021. In 2022, 4 to 5 replicates from 5 different F3 families were sown for a progeny test. For C11.2, the introgression line PS11.3 was crossed with the parental line VED, and recombinant F2 plants between the QTL flanking markers were selected (Supplementary Table S9b). A new line called 11PS carrying a smaller PS introgression was also generated. Sixty recombinant F2 plants, 4 11PS and 6 VED were sown and self-pollinated in 2021. In 2022, three to seven replicates belonging to 11 different F3 families were also sown for a progeny test.

Samples from fruit flesh and rind tissues were taken from every melon at fully ripe stage and were frozen at −80°C for further volatile analysis.

### DNA extraction and genotyping

Young leave tissue was used for DNA extraction by alkaline lysis [41] for quick genotyping. DNA from selected plants was also extracted according to the CTAB method [42] with minor modifications [14] for long-term storage.

The two IL collections were genotyped using a 96 SNPs set evenly distributed across the 12 chromosomes [10]. For the fine mappings, new SNP markers were developed within the QTL intervals based on resequencing data from the parental lines VED and PS (Supplementary Table S10). PCR Allele Competitive Extension (PACE) was performed in a LightCycler 480 Real-time PCR System (Roche Diagnostics, Spain) with PACE2.0 mastermix (3cr Bioscience, Essex, UK). Each well of a 384-well plate contained 2.5 μl of mastermix, 0.069 μl of the mix of primers (12 μM A1, 12 μM A2 and 36 μM C1) and 2.5 μl of 1:15 diluted DNA.

### Fruit quality phenotyping

Phenotyping of the fruits was done for fruit weight (FW), length (FL), width (FWI), perimeter (FP), area (FA), solid soluble content (SSC), flesh firmness (FIR), earliness of yellowing (EYELL), earliness of chlorophyll degradation (ECD), earliness of aroma production (EARO), earliness of abscission layer activation (EALF), abscission level (ABS) and harvest date (HAR), with the same parameters and units described in Pereira et al. (2021) [10] and Santo Domingo et al. (2022) [11]. Tomato Analyzer 3.0 was used for the fruit size measurements [43].

### Sample preparation for volatile extraction and GC–MS analysis

Frozen samples of rind and flesh tissues were ground up and stored at −80°C. Biological replicates were independently analysed by GC–MS. Chromatography vials were prepared as stated in Mayobre et al. (2021) [2], using 15 ppm (85.575 μg) of 3-hexanone as internal standard. Solid-Phase Micro-Extraction (SPME) was carried out with the same equipment and parameters, performing an untargeted analysis. Relative quantification to the internal standard was done. Information about the odor of the volatiles detected was extracted from FlavorDB (https://cosylab.iiitd.edu.in/flavordb/). Odor thresholds were extracted from the Leibniz Odorant Database (https://www.leibniz-lsb.de/en/databases/leibniz-lsbtum-odorant-database/odorantdb/).

### QTL detection and statistical analysis

Before the analysis, outlier removal was performed by calculating Z scores for every line and variable. The performance of this step was checked by doing a PCA as described by Fernández-Trujillo et al. (2018) [44]. A sample was considered an outlier when more than 10% of the VOCs had a Z score below −2 or above 2 (more than 2 standard deviations away from the mean of that group). Normality was checked with a Shapiro–Wilk test. QTL identification was done by comparison to the respective recurrent parental line. Comparisons were performed by Dunnett or Wilcoxon tests with Holm correction depending on the normality results. For fine mappings, Wilcoxon test with was applied for each volatile, marker by marker, comparing between recombinant lines with PS, heterozygous and VED alleles. Correlation tests were performed using the Pearson’s and Spearman’s rank correlation coefficient for normal and not normal data, respectively. The R software (v4.0.0) [45] with the RStudio interface (v1.1.463) [46] was used for graphs and statistical analysis with ‘ggfortify’, ‘Hmisc’, ‘corrplot’, ‘gplots’, ‘factoextra’, and ‘ggplot2’ packages. The *p*-value threshold was set to <0.05 for all the analyses. QTLs detected were named following previous criteria [2, 10, 11]. To define QTLs flanking positions, the mean of the physical position of the markers delimiting the introgression was used. The physical size of the introgressions was calculated using the melon reference genome v3.6.1 [47].

### Candidate gene selection

Functional annotation (v4.0) from the Melonomics website (https://www.melonomics.net/melonomics.html#) was used for candidate gene selection within the QTL interval. RNA-Seq data available in our laboratory from the parental lines VED and PS at different stages of fruit development and ripening (data in preparation) was used to support this selection on the most interesting regions, together with variant information for the fine mapping intervals (Supplementary Fig. S3, Supplementary Tables S6 and S7). An ortholog search was done in PLAZA 5.0 (https://bioinformatics.psb.ugent.be/plaza/versions/plaza_v5_dicots/) [48], and phylogenetic analyses were performed with MEGA11 [49], selecting for Neighbor-Joining (N-J) clusterization.

Genomic variants were identified for candidate genes of the fine mapping intervals by using resequencing data from the parental lines VED and PS [50]. Variants were also confirmed by Sanger sequencing on chromosome 6 candidate genes. For *MELO3C006443*, 5′-CGTTAAATCTGGATCAATCGCTAC-3′ was used as forward primer and 5′-GTACAAACGGCAGCATGAAA3′ as reverse. For *MELO3C006444*, 5′-TCCTGTCGTAATGGGTAATCG-3′ was used as forward and 5′-ATCGGATCCTGAAGCTTGTT-3 as reverse.

## Supplementary Material

Web_Material_uhae020

## Data Availability

The datasets generated and analysed during the current study can be found within manuscript and Supplementary information, with further enquiries being directed to the corresponding authors J.G.-M. and M.P.
