# Supplementary material for: Genetic regulation of volatile production in two melon introgression line collections with contrasting ripening behavior
Source: Hortic Res. 2024 Jan 16;11(3):uhae020. doi: 10.1093/hr/uhae020 (PMC10925849; doi:10.1093/hr/uhae020)
Supplement: Web_Material_uhae020 [file web_material_uhae020.zip › Supplementary FigS2.pdf]

>MELO3C006443_PS
MCTLEKRRNLFILTLTGDHDHRLNPSVIAALLEALSKVKSQASPGSVL**I**TTSHGKFFSNGFDLSWAHAAGSVSAAAERLHHMVQNFK
PVIAELLSLPMPTIAAISGHAAAAGFMLALSHDYLLMRSDRGVLYMSEVDLGLSLPDYFAALFKSKIGSSSVRRDVLMRGMKVKGEA
AVKMGIADS**A**YDGESGVMEAAVRLGEQLGARKWNGEPYAEIRKSLYPEISRLLGLPEKAISISKL

>MELO3C006443_VED
MCTLEKRRNLFILTLTGDHDHRLNPSVIAALLEALSKVKSQASPGSVL**V**TTSHGKFFSNGFDLSWAHAAGSVSAAAERLHHMVQNF
KPVIAELLSLPMPTIAAISGHAAAAGFMLALSHDYLLMRSDRGVLYMSEVDLGLSLPDYFAALFKSKIGSSSVRRDVLMRGMKVKGE
AAVKMGIADS**V**YDGESGVMEAAVRLGEQLGARKWNGEPYAEIRKSLYPEISRLLGLPEKAISISKL

>MELO3C006444_PS
MCSVEKRGKLFILTLTGDNEHWLNPSLMNSILNALSQLKAEARASPGSVLITTSQGKFFSNGLDLPWILSASSLSAARNRLNHMIQLF
KPLLAQLLCLPIPTIAVLPGHAAAAGLVLALTHDYLVMRSDRGVLYMSELDLGATLPDYFMALAKSKIGSSSVRRDVFLRGMKVRGE
TAVKMGVAES**E**HHGEDGVMEAAVRLGKELAARNW**D**GNAYAEIRKSLYPEICGLLGLTSKVITISKL

>MELO3C006444_VED
MCSVEKRGKLFILTLTGDNEHWLNPSLMNSILNALSQLKAEARASPGSVLITTSQGKFFSNGLDLPWILSASSLSAARNRLNHMIQLF
KPLLAQLLCLPIPTIAVLPGHAAAAGLVLALTHDYLVMRSDRGVLYMSELDLGATLPDYFMALAKSKIGSSSVRRDVFLRGMKVRGE
TAVKMGVAES**A**HHGEDGVMEAAVRLGKELAARNW**N**GNAYAEIRKSLYPEICGLLGLTSKVITISKL

| Gene | Substitution | Provean outcome | SIFT outcome |
|---|---|---|---|
| MELO3C006443 | I49V | Neutral | Tolerated |
| | A184V | Neutral | Tolerated |
| MELO3C006444 | E186A | Neutral | Tolerated |
| | D210N | Neutral | Tolerated |
