# Supplementary figures and images for: Genetic regulation of volatile production in two melon introgression line collections with contrasting ripening behavior
Source: Hortic Res. 2024 Jan 16;11(3):uhae020. doi: 10.1093/hr/uhae020 (PMC10925849; doi:10.1093/hr/uhae020)

(a)

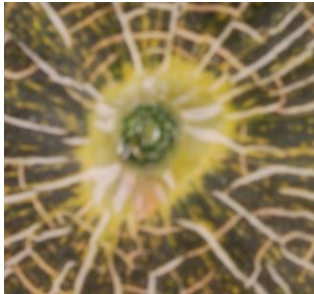

(b)

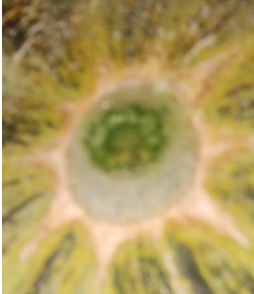

Supplement: Web_Material_uhae020 [file web_material_uhae020.zip › Supplementary FigS1.pdf]

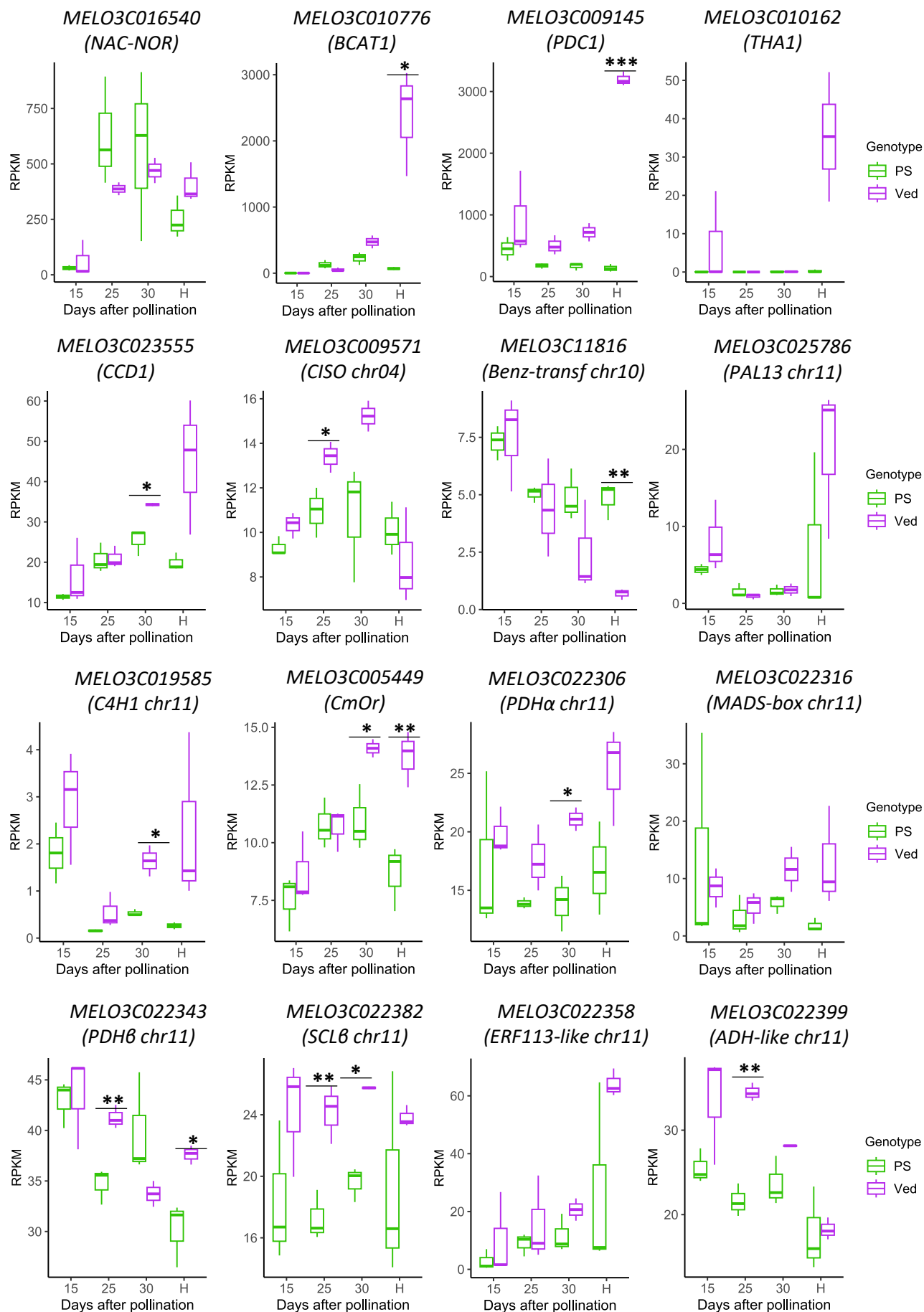

Supplement: Web_Material_uhae020 [file web_material_uhae020.zip › Supplementary FigS3_R1.pdf]
